# The Systematic Guideline Review: Method, rationale, and test on chronic heart failure

**DOI:** 10.1186/1472-6963-9-74

**Published:** 2009-05-08

**Authors:** Christiane Muth, Jochen Gensichen, Martin Beyer, Allen Hutchinson, Ferdinand M Gerlach

**Affiliations:** 1 Institute for General Practice, Johann Wolfgang Goethe University, Frankfurt/Main, Germany; 2 Institute for General Practice, University Hospital, Friedrich-Schiller-University, Jena, Germany; 3 School of Health and Related Research (ScHARR), University of Sheffield, Sheffield, UK

## Abstract

**Background:**

Evidence-based guidelines have the potential to improve healthcare. However, their de-novo-development requires substantial resources – especially for complex conditions, and adaptation may be biased by contextually influenced recommendations in source guidelines. In this paper we describe a new approach to guideline development – the systematic guideline review method (SGR), and its application in the development of an evidence-based guideline for family physicians on chronic heart failure (CHF).

**Methods:**

A systematic search for guidelines was carried out. Evidence-based guidelines on CHF management in adults in ambulatory care published in English or German between the years 2000 and 2004 were included. Guidelines on acute or right heart failure were excluded. Eligibility was assessed by two reviewers, methodological quality of selected guidelines was appraised using the AGREE instrument, and a framework of relevant clinical questions for diagnostics and treatment was derived. Data were extracted into evidence tables, systematically compared by means of a consistency analysis and synthesized in a preliminary draft. Most relevant primary sources were re-assessed to verify the cited evidence. Evidence and recommendations were summarized in a draft guideline.

**Results:**

Of 16 included guidelines five were of good quality. A total of 35 recommendations were systematically compared: 25/35 were consistent, 9/35 inconsistent, and 1/35 un-rateable (derived from a single guideline). Of the 25 consistencies, 14 were based on consensus, seven on evidence and four differed in grading. Major inconsistencies were found in 3/9 of the inconsistent recommendations. We re-evaluated the evidence for 17 recommendations (evidence-based, differing evidence levels and minor inconsistencies) – the majority was congruent. Incongruity was found where the stated evidence could not be verified in the cited primary sources, or where the evaluation in the source guidelines focused on treatment benefits and underestimated the risks. The draft guideline was completed in 8.5 man-months. The main limitation to this study was the lack of a second reviewer.

**Conclusion:**

The systematic guideline review including framework development, consistency analysis and validation is an effective, valid, and resource saving-approach to the development of evidence-based guidelines.

## Background

Evidence-based clinical practice guidelines have considerable potential to improve health care, and their international production has increased substantially over the past two decades [1-3]. However, the de-novo development of an evidence-based guideline requires considerable time, expertise and resources [4,5], the latter of which are limited, even in developed countries [6,7]. It was therefore suggested that their development should be based on the adaptation of existing guidelines [8].

A number of projects have sought to adapt or adopt existing guidelines [9-14], on topics ranging from HIV/AIDS [10] to the management of acute low back pain [14]. Each, in its own way, has highlighted the challenges of deriving new guidelines from existing material. Deficient development methods [15-20], subjectivity [21] and conflicts of interest [22,23] in the source guidelines may all lead to biases.

Additionally, implicit normativity has been shown to be inherent in guidelines – from the search for and critical appraisal of evidence, to its presentation, and the formulation of recommendations [24]. Contextual features arise from subject and financial constraints [25,26], ethical considerations and social influences [27-29], as well as practical necessities [30]. But presuppositions and values change, vary between cultures and healthcare systems, and are sometimes inconsistent, even within healthcare systems [30-34]. They may distort an adapted guideline to suit a different target context. Moreover, explaining and addressing context-specific normative considerations have been seen as a necessary condition for the successful implementation of guidelines [27].

All of these issues give rise to methodological challenges for adaptation processes. In recognition of this, the ADAPTE group [35] has proposed a seven-step framework which they call 'trans-contextual' adaptation. Fervers et al. [35] pointed out that it is of crucial importance to analyze the coherence between evidence and recommendations and to take culture and systems into account, but they did not provide the practical means to deal with this problem. In this paper we describe a new method that we have named the systematic guideline review (SGR). The SGR method was designed to take both methodological shortcomings and context-specific normative issues in source guidelines into consideration, in order to develop a valid guideline for a different target context in a resource saving manner.

The SGR was conducted as the first step in the development of a new guideline on chronic heart failure for use by family physicians in Germany. After having carried out the SGR, the resulting draft guideline underwent further development in accordance with institutional standards [36], which are out of scope of this paper: To ensure the involvement of stakeholders and target users, this comprised a multistage internal and external peer review, a multi-professional, interdisciplinary formal consensus that included a patients' representative (nominal group process), and a pilot testing phase. The final guideline was authorized by the German Society of General Practice and Family Medicine (DEGAM) and published in 2006 [37]. In our article we focus on the systematic guideline review.

## Methods

To develop an evidence-based guideline on chronic heart failure (CHF) for use in German primary care we defined, a priori, the key premises of the target guideline: the definition of the target condition, its epidemiology, the target setting, the need for change in healthcare, and the outcome parameters of interest.

**Figure 1:**
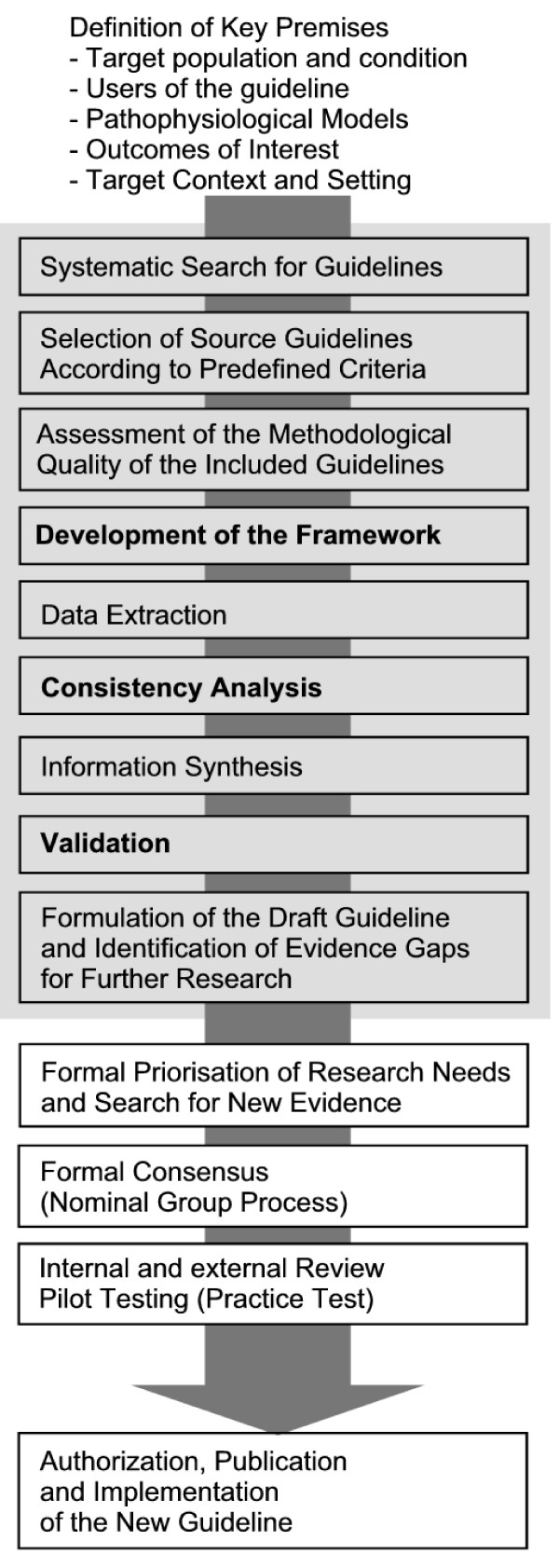
**Development of the Evidence-based Guideline on Chronic Heart Failure in Primary Care**. Gray Tagged: The Systematic Guideline Review.

We designed the SGR method comprising the nine steps grey tagged in FIGURE 1.

### Systematic guideline search

In March 2004 one of us (CM) performed a systematic literature search for existing guidelines in MEDLINE, The Cochrane Library, DARE, and HSTAT; combining controlled terms and free text, complemented by a comprehensive hand search of web-based resources (see Additional file 1, Tables W1 and W2) and in reference lists of the retrieved guidelines.

### Guideline Selection

Based on predefined criteria, two reviewers (CM, JG) independently appraised the retrieved guideline-documents for eligibility (yes or no). Discrepancies were resolved by consensus, and kappa-statistics were calculated for observer variability according to Cohen [38]. To be included a guideline had to be (1) dedicated to adult patients with chronic heart failure (CHF), (2) evidence-based, i.e. evidence levels (and/or a grading) were reported in the majority of recommendations with a clear link to supporting evidence, (3) released in the year 2000 or later, and (4) published in English or German.

A guideline was excluded, if it referred exclusively to pediatric patients, isolated right heart failure, special or inpatient care, or no information on development methods was given (at least, an institutional standard of the developing organization had to be provided). Guidelines with a focus only on particular aspects of heart failure management (e.g. guidelines on echocardiography) were excluded from the SGR.

### Quality appraisal of guidelines

The quality of the guideline was assessed using the standardized Appraisal of Guidelines Research and Evaluation (AGREE) instrument [39,40]. AGREE consists of 23 items which rate the various dimensions of guideline quality by means of four-point Likert scales. The items are organized into six independent domains. Domain scores are calculated by summing up the scores of the items per domain and are standardised as a percentage of the maximum possible score for that domain. [39]. There is no universal agreement on specific cut off scores to identify high quality guidelines, and differing approaches are applied [41,42]. To rate the overall guideline quality we defined a median target and result score per guideline (calculated as the median of the standardized domain scores over all domains) of 0.5 or above as indicating good quality.

AGREE is suggested for multiple raters. The appraisal is based on the consideration of the whole guideline and is therefore of wider scope than the evidence review and synthesis reported here. Nevertheless, it does include any recommendations on diagnosis, monitoring, or prevention and the proposed multiple rater scheme is thus rather time-consuming. Because we had only limited resources available to us, we performed a single rating (CM) for this step of the guideline development process.

### Framework and data extraction

To build a framework of relevant issues, clinical questions from the included guidelines were derived by one reviewer (CM), appraised for their relevance and prioritized in terms of the target context by three others (JG, MB, FMG). For each clinical question the data were extractedinto evidence tables of a standardized format which included recommendation(s), evidence level(s), grading, critical appraisal of evidence, and cited sources.

### Consistency analysis

One reviewer (CM) systematically compared guideline recommendations for every clinical question for their external consistency (e.g. between guidelines), and their reported evidence. We categorized our findings into six types (Table 1): four types of consistency and two types of inconsistency (major and minor). The categories were built on both dimensions (external consistency and evidence base) to support a further action plan for guideline development: low levels of evidence as well as inconsistencies between guidelines indicated the need for further research. The classification system was drafted by two of us (CM and MB) and consented with the others (JG, AH, FMG). One reviewer (CM) synthesized the information (recommendations, their consistency, as well as the reported underlying evidence from the included guidelines) in a preliminary draft.

**Table 1:** Categories of Consistency and Inconsistency

**Type**	**Definition**	**Further Action Plan for Guideline Development**
**Consistency**		

(1)	**Recommendations are consistent **in content, evidence level and grading, and **based on a *large *body of high level evidence **(e.g. multiple primary or secondary studies of high internal and external validity)	Verification of cited sources with highest evidence level, and update searches, if necessary

(2)	**Recommendations are consistent **in content, evidence level and grading, and **based on a *small *body of high level evidence **(e.g. a single or a few primary studies of high internal and external validity)	Verification of cited sources, further research on safety aspects in particular, and update searches

(3)	**Recommendations are consistent **in content, evidence level and grading, and **based on **evidence from studies of **low level evidence **(e.g. studies with design-related biases or where methodological flaws reduce internal or external validity) **or based on expert opinion **(where evidence is lacking)	Further research on evidence

(4)	**Recommendations are consistent in content**, but **evidence levels and grading conflict**	Verification of cited sources, and update searches

**Inconsistency**		

(A)	**Recommendations are completely inconsistent**, neither a mainstream trend nor even a common denominator can be identified	Further research on evidence

(B)	**Recommendations are consistent in the majority **of guidelines, but **differing **or even conflicting **recommendations are to be found in a minority**	Verification of cited sources to decide whether further research is necessary, and update-searches

### Validation

For a critical re-appraisal of whether recommendations were supported by valid study results, the most relevant evidence cited in the included guidelines was selected by applying the highest levels of evidence from the Oxford Centre for Evidence-Based Medicine [43]. Predominantly systematic reviews with or without meta-analyses were re-evaluated, along with clinical studies of an appropriate design when secondary publications did not provide the desired evidence. The selected studies were re-evaluated for their internal and external validity. For the assessment, checklists from the German Working Group on Health Technology Assessment (see Additional file 1, non-scoring standardized instruments, Table W3) were used, since one of us (CM) was trained and experienced in its use [44]. In particular, the studies were investigated for inherent bias, which might have influenced results, and for the applicability of their results (in accordance with the defined key premises of the target guideline: study population representative of target population, clinically relevant outcomes reported in the study, setting of the study appropriate with regard to the target setting).

### Draft guideline

Finally, the evidence and recommendations were synthesized in a draft of the target guideline, and the results and possible methodological flaws of the included source-guidelines, and the re-assessed studies, were discussed. For specified clinical questions without identified high-quality evidence and for the necessary update-searches to ensure the actuality of the guideline, a structured list was formed to define the need for further research.

## Results

### Literature Search and Selection

**Figure 2:**
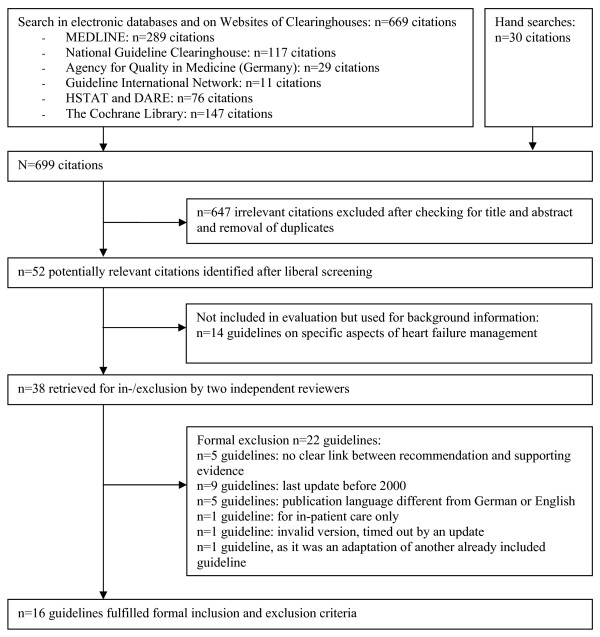
**Results of the Systematic Guideline Search (Flowchart)**.

Our literature search resulted in 699 citations, of which 52 were potentially relevant. A total of 16 evidence-based guidelines met our inclusion criteria ( FIGURE 2). The inter-observer variability was excellent (one discrepancy; κ = 0.95). Of the included guidelines (Table 2), five were from Germany [45-49], four from the U.S. [50-54], two each from Canada [55-57] Australia & New Zealand [58,59], and one each from Finland [60], the U.K. [61], and Europe [62]. The context and setting of the guidelines varied: five guidelines were prepared for a specific healthcare setting (e.g. ambulatory care in a health maintenance organization) [48,51-54,57], one guideline was directed towards CHF management throughout Europe [62], one guideline did not specify any target group [49], and the remaining guidelines referred to national healthcare. The scope of the guidelines varied considerably (all addressed pharmacotherapy in heart failure, 13/16 non-pharmacological therapy, 10/16 diagnostics), as did the size of the guideline development groups (2 to 33; median 13), the number of citations (24 to 573; median 132), the volume of the full text version (16 to 163 pages in the long versions), and the quantity and layout of additional tools. A complete list of excluded guidelines, together with the reasons for exclusion, is provided as additional web-material (see Additional file 1, Table W4).

**Table 2:** Characteristics of Included Guidelines

**Source**	**Organisation, Country of Origin**	**Covered Scope^†^**	**No. of Authors**	**Literature Search: Period/Databases**	**No. of References**
ACC/AHA 2001 [50]	American College of Cardiology/American Heart Association, U.S.A.	D, T	14	Period not specified MEDLINE, EMBASE	573

AKDAE 2001 [45]	Drug Commission of the German Medical Association, Germany	P	Not specified	No systematic search	216

CCS 2001 [56], 2002/3 [55]	Canadian Cardiovascular Society, Canada	D, T	Not specified	Period not specified MEDLINE	2001: 79; 2002/3: 42

DGK 2001 [46]	German Cardiac Society, Germany	T	2	1990–2000; Databases: not specified	213

DieM 2003/2004 [47]	Institute for Evidence-Based Medicine, Germany	T	3	Period not specified (Last 03/2003); MEDLINE, Cochrane Library, „Best Evidence"	42

Duodecim 2004 [60]	The Finnish Medical Society Duodecim, Finland	D, T	Not specified	Period not specified (Last 03/2004); DARE, „Best Evidence"	Ca. 50^††^

DVA & VHA 2002 [51]	Department of Veterans Affairs & Veterans Health Administration, U.S.A.	P	14*	Update search 01/2001 to 11/2002; MEDLINE	197

ESC 2002/2001 [62]	European Society of Cardiology, (Europe)	D, T	18	Not specified	196

ICSI 2003 [52]	Institute for Clinical System Improvement, U.S.A.	D, T	11	Not specified	Ca. 50

LLGH 2003 [48]	'Leitliniengruppe' (Group of Family Physicians in Hesse), Germany	(D), T	2	Period not specified MEDLINE	72

NHF/Austr & SANZ 2002 [58]	National Heart Foundation of Australia and Cardiac Society of Australia & New Zealand	D, T	33	Not specified	143

NHF/NZ 2001 [59]	The National Heart Foundation of New Zealand	D, T	19	Period not specified (Last 04/2000); MEDLINE	44

NICE 2003 [61]	The National Collaborating Centre for Chronic Conditions/National Institute for Clinical Excellence, United Kingdom	D, T	15**	Start of the Database until 09/2002; MEDLINE, EMBASE, CINAHL, PsycINFO, AMED, Cochrane Library, EconLit	347^‡^

OPOT 2000 [57]	Ontario Program for Optimal Therapeutics, Canada	P	Not specified	Not specified	24

UM 2001 [53,54]	University of Michigan, U.S.A.	D, T	6	1994 – 02/1998, + hand searches until 2001; MEDLINE	50

UWH 2001 [49]	Faculty of Medicine, University Witten/Herdecke, Germany	D, T	5 + 2^‡‡^	Not specified	157

^†^Limitation on recommendations with evidence levels and/or grading; ^††^Ascertainment possibly incomplete because of document structure (internet-based version with links to other documents); *Supported by 13 members of The Medical Advisory; **Support from 14 members of the Guideline Reference Group; ^‡^Additional citations in evidence tables not counted; ^‡‡^Additional advisors from specialized care; ***Abbreviations: ***D – diagnostics, (D) – diagnostics partly, T – therapy, P – pharmacotherapy only

### Appraisal of the Methodological Quality

Five guidelines [48,50-52,61] were found to be of high quality according to our criteria. Methodological quality varied broadly between both guidelines (ranging from 0.2 to 0.7) and domains (Table 3) and the key results of the most important domains for the SGR were as follows: In domain 3 "rigour of development" 7/16 guidelines scored well: 13/16 used systematic methods in their search for evidence; 12/16 explained methods for formulating their recommendations – two of which included formal consensus techniques; 12/16 provided information on the update process – seven in detail. All guidelines discussed health benefits, side effects, and the risks of applying most (or at least the key) recommendations, 11/16 reported on a formal external peer review process. The main weakness appeared in the description of how the evidence was selected: only 4/16 guidelines reported explicit criteria. In domain 6 "editorial independence" four guidelines scored 0.0 (no information about funding source, no financial disclosures for their group members), one guideline scored 1.0 (full information on financing, and declarations on potential conflicts of interest), the remainder in between.

**Table 3:** Summarized Methodological Quality of Included Guidelines (AGREE Instrument, Standardized Domain Scores in Brackets)

	**Domain 1** 'Scope and Purpose' 3–12 Points	**Domain 2** 'Stakeholder Involvement' 4–16 Points	**Domain 3** 'Rigour of Development' 7–28 Points	**Domain 4** 'Clarity and Presentation' 4–16 Points	**Domain 5** 'Application' 3–12 Points	**Domain 6** 'Editorial Independence' 2–8 Points
**Median**	6.5 (0.39)	8.5 (0.38)	17 (0.48)	12.5 (0.71)	4.5 (0.17)	4 (0.33)

**Range**	4–11 (0.11 – 0.89)	4–12 (0 – 0.67)	11–23 (0.19 – 0.76)	10–16 (0.5 – 1.0)	3–9 (0 – 0.67)	2–8 (0 – 1.0)

### Framework Development and Data Extraction

In a stepwise approach we derived 27 clinical questions for the framework of our target-guideline. Some questions were partly complex, i.e. they comprised more than one research question (e.g. 'Should patients be encouraged to do exercise training, and if so, what intensity and duration should be aimed for?'). To extract the data we entered all relevant information into evidence tables in the above mentioned format.

### Consistency Analysis

Within the framework we identified 35 recommendations. Of these, 25 recommendations were consistent, nine were inconsistent, and one was not rateable (derived from a single guideline). Of 25 consistencies, seven were based on strong evidence (types 1 and 2), 14/25 on weak evidence or expert consensus (type 3), and 4/25 were consistent in content, but had different evidence levels (type 4). Only one inconsistent recommendation (a minor inconsistency about salt and fluid restriction) was based on expert consensus, the remaining inconsistencies differed in their evaluation of the empirical findings (types A and B).

While we found type 1-, 2-, and 4-consistencies mainly in recommendations on pharmacotherapy (Table 4), type 3-consistencies addressed all kinds of clinical questions, from diagnostics to education, monitoring, and the definition of interfaces to specialized ambulatory and in-patient care.

**Table 4:** Results of Consistency Analysis and Validation

**Type**	**Clinical Question**	**Consistency Analysis^†^**	**Validation**	**Comment**
**Consistencies**				

**(1)**	Use of ACE inhibitors in systolic CHF, all NYHA classes (incl. asymptomatic patients NYHA class I, with or without history of myocardial infarction)	16/16 'recommended'	Partly justified	Benefit was shown for symptomatic patients (all outcomes incl. mortality), in asymptomatic patients NYHA class I: improvement of prognosis and morbidity, but no evidence for a mortality reduction (see text)
	
	Use of beta-blockers in systolic CHF, NYHA I post myocardial infarction	11/11 'recommended'	Completely justified	Cited sources provided the reported evidence in form and content
	
	Use of beta-blockers in systolic CHF, NYHA II-III	16/16 'recommended'	Completely justified	Cited sources provided the reported evidence in form and content

**(2)**	Use of aldosterone antagonists in systolic CHF, NYHA III/IV	16/16 'recommended'	Justified	Cited sources provided evidence on effectiveness; further research is needed on safety (see text)
	
	Use of digoxin in systolic CHF with tachyarrhythmia	15/15 'recommended'	Partly justified	Evidence level were revised (see text)
	
	Control of hypertension in diastolic CHF	2/2 'recommended'	Not justified	Insufficient evidence, further research is needed (see text)
	
	Use of anticoagulants in patients with the combination of CHF and atrial fibrillation and/or a history of thromboembolism	12/12 'recommended'	-	No re-assessment: recommendations referred to atrial fibrillation (out of scope in the target guideline)

**(4)**	Exercise Training	13/13 'recommended'	-	No re-assessment: evidence was to be found in a newly identified meta-analysis [63]
	
	Diuretics in systolic CHF, NYHA II-IV	14/14 'recommended'	Partly justified	Evidence level was revised (see text)
	
	Use of hydralazine plus ISDN in ACE inhibitor-/ARB-intolerant patients	10/10 'recommended'	-	No re-assessment: no market availability for the fixed combination in the target context
	
	Harmlessness of long-acting dihydropyridines	7/7 'recommended'	Partly justified	Evidence levels not justified; evidence insufficient, further research is needed

**Inconsistencies**				

**(B)**	Salt and fluid restriction (varying quantification)	9/10 'recommended', 1/10 'not recommended'	-	No validation: recommendations based on expert consensus

	Beta-blockers in clinical stable systolic CHF, NYHA IV	13/15 'recommended', 2/15 'not recommended'	Majority was justified, minority was rejected	Positive recommendations completely justified, negative recommendations based on insufficient evidence
	
	Beta-blockers in all systolic CHF, NYHA I – no matter whether post myocardial infarction or non-ischemic genesis	7/8 'recommended', 1/8 'consideration recommended'	Majority was not justified, minority was accepted	No evidence for strong recommendation (see text)
	
	ARB in ACE intolerant patients	15/16 'recommended', 1/16 potentially harmful therapy	Majority justified, minority rejected	Positive recommendations justified, negative recommendations based on insufficient evidence

^†^Numerical proportion of the mandating to guidelines which covered the scope and reported evidence levels and graded their recommendation. Type-3-consistencies – based on weak evidence – and type-A-inconsistencies are not listed in this table, as they were not included in the validation procedure but needed further research for evidence (a list is provided as additional web-based material, TABLE W5).

Among nine inconsistencies we classified three as major inconsistencies (type A): (1) The use of brain natriuretic peptide testing in patients when CHF is suspected (Table 5); (2) Angiotensin II receptor blockers (ARB) in addition to ACE inhibitors and beta-blockers (triple-therapy); and (3) ARB in combination with ACE inhibitors in beta-blocker-intolerant patients (substitution of beta-blockers).

**Table 5:** Case Study about Brain Natriuretic Peptides (BNP) in the Diagnosis of Heart Failure*

**Results from the SGR: **The recommendations in the source guidelines on the use of BNP tests in patients suspected of heart failure showed a major inconsistency (type A): The test was treated in 7/16 guidelines; recommendations differed completely in content (2/7 'not recommended', 3/7 'recommended under certain circumstances', 1/7 'recommended in every case', 1/7 'recommended ruling out CHF before an echocardiogram'), and in grading.
**Further research: **We conducted a systematic review of the diagnostic accuracy of this test in primary care. We did not find strong evidence in favour of its use in this setting (most studies were undertaken after referral which implies a potential spectrum bias [64,65], a clear cut-off was not defined, study results were inconsistent, in particular for concomitant diseases and medication) [66-69], supported by two subsequently published systematic reviews [70,71]. The consensus panel agreed not to recommend the test in our target guideline.

**Discussion: **Inconsistent recommendations in source guidelines may be due to (i) *methodological shortcomings *(recommendations were not setting-specific in 6/7 guidelines that addressed both primary and secondary care; literature searches were stated to be comprehensive in only 2/7 guidelines), or to (ii) *potential conflicts of interests *(4/7 guidelines did not provide any financial disclosures for the authors). Moreover, (iii) *contextual influences *may have guided the recommendations, such as availability (1/7 guidelines recommended the test, as echocardiograms are not widely available), access (BNP tests have market approval throughout Europe but costs are reimbursed by public funding – e.g. in the U.K. – or privately, e.g. in Germany), or intended resource allocation (1/7 guidelines restricted access to the more expensive echocardiogram, as CHF had to be ruled out by BNP and/or electrocardiogram before the referral). Last but not least the BNP test is an emerging technology where typically only limited information of its benefit is available, and initial studies show predominantly optimistic results [72]. Variations in the adoption of a new (healthcare) technology from one country to another, and also from one physician to another are shown to be large, and 'inextricably interwoven' with culture [72]. We know that more highly trained and committed physicians in the community tend to be 'early adopters' [72], and that specialists are less conservative than generalists [72,73]. It might be that the selection of guideline developing groups and their attitudes influenced the decision to include the BNP test.

*This test was developed to distinguish between heart failure and other conditions that show typical symptoms and signs in a patient. In this paper we use BNP as a synonym for itself and others such as NT-proBNP.

### Validation

Seventeen partly complex recommendations were categorized as consistency types 1, 2, 4 (n = 11), and inconsistency type B (n = 6) (Table 4). They were further examined in the validation procedure, while type-3-consistencies and major inconsistencies indicated the need for further search for new evidence rather than validation (see Additional file 1, for a complete list of type-3-consistencies and type-A-inconsistencies see Table W5).

To address the clinical questions under investigation the guidelines cited 309 documents, of which we considered 21 studies for re-assessment: 14 systematic reviews (SR) with or without meta-analyses, six RCTs and one post-hoc subgroup analysis of an RCT, since this study was linked to a warning sign in one guideline. The reasons for preclusion of the remaining studies are given below (Table 6). The validation procedure (Table 4) showed most of the recommendations to be justified by the cited evidence sources. Nevertheless, we found incongruity – three concise examples are given below:

**Table 6:** Selection of Studies for Re-assessment

**Publication Type**	**No. of Cited**	**No. of Excluded**	**Reason for Preclusion**
**Systematic Reviews/Meta-analyses (SR)**	36	13	Results outdated by more recent SR
		
		3	SR reported surrogate outcomes where clinical outcomes were available
		
		2	SR did not contain target population
		
		4	SR was out of the scope of the target guideline

**Randomized Controlled Trials (RCT)**	170	132	RCT included in re-evaluated SR
		
		8	Results on surrogate outcomes where clinical outcomes were available
		
		12	Set aside for further comprehensive research
		
		12	RCT was out of the scope of the target guideline

**Traditional Reviews, Editorials**	33	33	Provided no systematic evidence

**Miscellaneous Clinical Studies**	70	69	Study design and/or sample size N<50 were not expected to provide strong evidence; further search for high-level evidence was seen to be more effective than re-appraisal

**Total**	309	288	

#### (1) Incongruity due to a lack of evidence

All included guidelines recommended the use of ACE inhibitors in all NYHA classes including asymptomatic patients. While 11 guidelines designed differentiated clinical outcomes as therapeutic goals in asymptomatic patients (e.g. improvement in prognosis and hospitalization), three guidelines ranked evidence-level highest for a mortality reduction in this subpopulation, and two guidelines used ambiguous formulations. We found no evidence from the cited studies for a mortality reduction in asymptomatic patients, since this subpopulation was underrepresented in RCTs. Evidence was found only when we identified an health technology assessment report in further searches [74]. Comparable incongruity was found in recommendations for beta-blocker therapy in asymptomatic patients without prior myocardial infarction.

#### (2) Incongruity due to the ambiguous use of evidence levels

Fourteen guidelines recommended the use of diuretics, reported evidence levels and graded their recommendations: in 2/14 diuretics were recommended to reduce mortality and morbidity in CHF. The supporting evidence was ranked highest and linked to a systematic review including meta-analysis (published in 2002) [75], which was not accepted by another guideline on the basis of its equivocal methodological quality. In 12 guidelines the main therapeutic goal of diuretics was to control fluid retention: 7/12 ranked the evidence highest, 2/12 second highest and 2/12 lowest (expert opinion). We re-appraised the meta-analysis of 17 small sample sized RCTs and confirmed concerns about its methodological quality (no discussion of the methodological quality of included studies, homogeneity assumption after the χ^2^-test detected no heterogeneity despite high clinical heterogeneity between the studies, no sensitivity analyses to test the robustness of the results). In conclusion, the evidence level remained unclear: formally, multiple RCTs have shown positive effects on (different) clinical outcomes of diuretics, but the methodological quality of the meta-analyses (and the RCTs themselves) give a high probability of biased results.

#### (3) Other kinds of incongruence

The evaluation of pharmacotherapy in the source guidelines often focused on treatment benefits and underestimated the risks, such as of adverse events, following the combination of ARBs and ACE inhibitors, or the risk of hyperkalemia following the use of aldosterone antagonists, as recently shown [76,77].

### Draft guideline and formulation of the needs for further research

We summarized the SGR results in a draft guideline for the following steps in development and formed a list of specific clinical questions for further research comprising all type-3-consistencies, type-A-inconsistencies and incongruity from the validation process. To adjust the research needs to the given resources these research questions were formally prioritized for the search for new evidence by representatives from the targeted user groups after conducting the SGR (outside the scope of this paper).

### Resources

Our methodological concept of the systematic guideline review had to be planned with exceedingly limited resources for the whole project (the budget was € 75.000), but was successfully completed after 8.5 man-months. Starting in January 2004 with the development of the methods concept and systematic searches for guidelines, the first part of the SGR was finished by one researcher (part time: 75%) by the end of June 2004, accounting for 4.5 man-months. From July 2004 to February 2005 the validation procedure was conducted and the draft guideline was written by one researcher (50%), accounting for the remaining four man-months.

## Discussion

Our study addressed the need for a high-quality evidence-based guideline to manage patients with CHF in the German primary care context. Internationally, several high-ranking guidelines were/are already in existence, but adaptation methods have not been established yet [78,79]. The guidelines included in this study were heterogeneous in many respects, such as origin, coverage, and the extent of appraised evidence. Nevertheless, the analysis of these guidelines by means of the systematic guideline review enabled us to develop a new evidence-based guideline on a complex condition with comparatively limited resources. In our opinion three steps in the SGR were responsible for resource saving in terms of net effort, as well as for improving the validity and transparency of the target guideline: (1) construction of a framework, (2) consistency analysis, and (3) validation.

### Framework Building

CHF is a syndrome with a number of distinct clinical presentations [80]. 'Asking the right questions and asking them right' was named as the core task of guideline development [81], and it is essential for both appraisal of the identified evidence [82-84] and the practical needs to be met by a guideline. Often it is a time-consuming and un-transparent process, and there is inevitably a trade-off between depth and breadth of scope in accordance with development resources [85]. In our approach we extracted questions that were seen as relevant by other guideline development groups, which we then prioritized and refined to assure relevance to our setting. Thus it was a time-saving, systematic and (by publishing it in the methods report) a transparent process.

### Consistency Analysis

We carefully compared recommendations and the judgments of underlying evidence reported in the guidelines. Like Kulig et al., we found good overall (external) consistency [86]. Nevertheless, many recommendations were based only on expert consensus or weak evidence. We categorized our findings to bring to light these 'grey zones of clinical practice' [73], but also fields of controversy in CHF management such as BNP testing (Table 5). Our classification supported the specification of questions for further research, and their transparent prioritization. When supported by update-searches to ensure the timeliness of our target guideline, the SGR prevents unnecessary repetition of extensive evidence searches and appraisals: evidence-based consistencies (type-1-consistencies) allow a focused re-appraisal of the most important evidence sources. In type 2-consistencies further research may concentrate on safety aspects, as controlled studies are not generally sufficient to identify risks or their frequency [72].

### Validation

We critically re-appraised the most important evidence sources cited in the guidelines to assess whether design, study-population and results were able to support the recommendations in our setting. Certainly, the vast majority of recommendations was justified by empirical findings, but some were not. Examples such as strong recommendations for drug therapy in asymptomatic patients, which were not sufficiently based on the highest level of evidence (not being obsolete, but solely based on the consensus based extrapolation of studies with patients of higher NYHA-classes) shed a light on methodological shortcomings even in those guidelines that were of high quality according to the AGREE appraisal. Our reported findings fit in well with recently published studies: McAlister et al. [87] have shown that less than one-third of treatment recommendations (and less than half of those citing RCTs in support of the advocated treatment) were based on high-quality evidence in national evidence-based guidelines for common conditions, in particular when external validity was adequately taken into account. Also Watine et al. [42,88] demonstrated that guideline quality was not necessarily associated with valid recommendations.

Some of our judgments might seem overcritical to the reader. But the questions on how to compare and combine different sorts of evidence (e.g. benefits and risks) or conflicting study results reflect not only the under-use of current approaches to grade the evidence (e.g. the GRADE- or CHEP-system) [89,90] but also epistemological problems in evidence-based medicine [91,92]. Moreover, they point out that the concept of effectiveness is inherently interwoven with normative values [93], which vary depending on cultural context.

In comparison to adaptation procedures – e.g. the most ambitious approach by Fervers et al [35] – our SGR method differs mainly in the systematic consistency analysis and the validation procedure. Other steps are comparable (the systematic search for, selection and critical appraisal of the guideline) or necessary requirements for the SGR (data extraction and information synthesis). Fervers et al [35] emphasized the importance of proving coherence between evidence and recommendations and their applicability and acceptance to the target context [79]. We have shown that consistency analyses and the validation procedure of the SGR demonstrate coherence and further steps in guideline development proved applicability and acceptance.

Furthermore, our SGR method contributed to resource saving: The total costs for the guideline development (incl. the consensus process) were about € 75,000. Though the following examples do not allow a head-to-head comparison, they describe the context: for the 1997 Scottish guideline on 'The management of mild, non-proteinuric hypertension in pregnancy' the total costs were given as £ 66,809 (in those days about € 95,000) [94]; the average expenditure on evidence-based guidelines of the Agency for Health Care Policy and Research (AHCPR) in the nineties was approximately USD1 Million per guideline [95], and at the beginning of 2000 the Agency for Health Quality and Research (AHRQ) spent about USD 250,000 to conduct a systematic review [96].

Our study had some limitations such as the lack of a second review in data extraction and further steps of appraisal. Therefore, a certain observer bias cannot be ruled out. Our budget allowed only a single AGREE appraisal. Fortunately, we were able to compare our results with another working group of the German Agency for Quality in Medicine (AQuMed/ÄZQ) (two appraisers), which carried out a quality appraisal of the same guidelines subsequently and independently of us. Their results were quite similar with a slightly better rating than ours (see Additional file 1, Table W6). Furthermore, we disclosed all SGR material (e.g. evidence tables) to the participants in the consensus process and to the public (methods report in German is available online: ) to diminish the potential effects of an observer bias on the target guideline.

Also, due to limited resources, we confined the publication languages to English and German, which may lead to a system-related bias (to focus on a specific healthcare system which may vary from the target context). However, this bias is less probable in our study, since we included guidelines from several countries, differing in funding and reimbursement [97,98], availability and accessibility [99,100] of healthcare.

Further, our findings on the resource saving effect of the SGR have to be interpreted within the context of this study: our paper reports on an uncontrolled N = 1 group study and the resource data were collected retrospectively.

Nevertheless, our findings in the SGR were relevant to the recommendations in our guideline, assured their validity, contributed to its transparency, highlighted fields of implicit normativity to prepare an open discourse during the later stages of guideline development (the peer reviews, the consensus process, and the pilot testing), and saved resources by avoiding unnecessary redundancies in the literature search and appraisal. Further studies should be carried out with the involvement of a second reviewer to avoid observer bias.

## Conclusion

The systematic guideline review method is a valid means of making use of existing guidelines, and allows a systematic approach to the development of a new guideline. A systematic guideline search aims at the inclusion of guidelines from different healthcare systems, and a systematic comparison of recommendations brings to light both mainstream recommendations and controversies, but also the grey zones of clinical practice. A careful re-evaluation of the most relevant evidence sources assures validity. In the trade-off between breadth and depth, the SGR allows reasonable and transparent prioritization in order to concentrate the development resources on the most important questions for further research. The SGR helps to highlight fields of implicit normativity in guidelines and prepares them for an open discourse within the target context. It abbreviates the initial full evidence review stages, and helps to avoid unnecessary repetition of high-quality research by other guideline authors. In our example it allowed the development of a guideline on the complex clinical issue of chronic heart failure with comparatively small resources. Further studies will have to confirm our results to develop a valid guideline by means of the SGR.

## Competing interests

The authors declare that they have no competing interests.

## Authors' contributions

CM had full access to all the data in the study and takes responsibility for the integrity of the data and the accuracy of the data analysis. She designed the study, acquired, analyzed and interpreted the data and drafted the manuscript. JG, MB and FMG were involved in the study concept and design, supported data acquisition, analysis and interpretation and the critical revision of the manuscript for important intellectual content. AH substantially contributed to the data analysis and interpretation and critical revision of the manuscript for important intellectual content. All authors read and approved the final manuscript.

## Pre-publication history

The pre-publication history for this paper can be accessed here:



## Supplementary Material

Additional file 1 **Supporting Web-based Material**. The file contains the following tables, as mentioned in the text: – List of websites consulted in hand searches: International Organizations (Table W1). – List of websites consulted in hand searches: National Organizations (Table W2). – Checklist by the German Working Group on Health Technology Assessment: an example for systematic reviews with/without meta-analyses (Table W3). – List of excluded guidelines with the reason for preclusion (Table W4). – Results of Consistency Analyses: List of Type-3-Consistencies and Type-A-Inconsistencies (Table W5). – Comparison of different quality appraisals for the included guidelines (Table W6). Click here for file
