# Supplementary material for: The Systematic Guideline Review: Method, rationale, and test on chronic heart failure
Source: BMC Health Serv Res. 2009 May 8;9:74. doi: 10.1186/1472-6963-9-74 (PMC2698839; doi:10.1186/1472-6963-9-74)
Supplement: Additional file 1 — Supporting Web-based Material. The file contains the following tables, as mentioned in the text: – List of websites consulted in hand searches: International Organizations (Table W1). – List of websites consulted in hand searches: National Organizations (Table W2). – Checklist by the German Working Group on Health Technology Assessment: an example for systematic reviews with/without meta-analyses (Table W3). – List of excluded guidelines with the reason for preclusion (Table W4). – Results of Consistency Analyses: List of Type-3-Consistencies and Type-A-Inconsistencies (Table W5). – Comparison of different quality appraisals for the included guidelines (Table W6). [file 1472-6963-9-74-S1.doc]

Table W1: List of websites consulted in hand searches: International Organizations

| Abbreviation | Name and Country of the Institution, Internet-Address |
| --- | --- |
| ACC | American College of Cardiology (U.S.A.), [http://www.acc.org](http://www.acc.org/) |
| AETMIS | Agence d'Evaluation des Technologies et des Modes d'Intervention en Santé (Canada), <http://www.aetmis.gouv.qc.ca/fr/> |
| AHFMR | Alberta Heritage Foundation for Medical Research (Canada), <http://www.ahfmr.ab.ca/> |
| AHRQ [AHCPR] | Agency for Healthcare Research and Quality, [http://www.ahrq.gov](http://www.ahrq.gov/) (formerly Agency for Health Care Policy and Research (U.S.A.)) |
| AMA | Alberta Medical Association (Canada), <http://www.albertadoctors.org/> |
| CAHTA | Catalan Agency for Health Technology Assessment and Research (Spain), <http://www.aatrm.net/ang/ang.html> |
| CCOHTA | Canadian Coordinating Office for Health Technology Assessment (Canada), <http://www.ccohta.ca/> |
| CDHSH | Commonwealth Department of Human Services and Health (Australia), [www.health.gov.au](http://www.health.gov.au/) |
| CEDIT | Comité d’Evaluation et de Diffusion des Innovations Technologiques (France), <http://cedit.aphp.fr/english/index_present.html> |
| CTFPHC | Canadian Task Force on Preventive Health Care (Canada), <http://www.ctfphc.org/> |
| Duodecim | Leitlinienseite von The Finnish Medical Society Duodecim (Finland), <http://www.ebm-guidelines.com/home.html> |
| GIN | Guideline International Network, [http://www.g-i-n.net](http://www.g-i-n.net/) |
| GR | Gezondheidsraad (Netherlands), <http://www.gr.nl/> |
| HHS | Unites States Department of Health and Human Services (U.S.A.), [http://www.hhs.gov](http://www.hhs.gov/) |
| ICSI | Institute for Clinical Systems Improvement (U.S.A.), [http://www.icsi.org](http://www.icsi.org/) |
| INAHTA | International Network of Agencies for HTA (the former international organization for health technology assessment, today HTAI – Health Technology Assessment International), [http://www.inahta.org](http://www.inahta.org/) |
| ITA | Institut für Technikfolgen-Abschätzung (Austria), <http://www.oeaw.ac.at/ita/hta/> |
| MSAC | Medical Services Advisory Committee (Australia), <http://www.msac.gov.au/> |
| NCCHTA | National Coordinating Centre for Health Technology Assessment (U.K.), <http://www.hta.nhsweb.nhs.uk/> |
| NGC | National Guideline Clearinghouse (U.S.A), [http://www.guideline.gov](http://www.guideline.gov/) |
| NHG | Nederlands Huisartsen Genootschap (Netherlands), [http://nhg.artsennet.nl](http://nhg.artsennet.nl/) |
| NHS | National Health Services (U.K.), [http://www.nhsdirect.nhs.uk](http://www.nhsdirect.nhs.uk/) |
| NHSC | National Horizon Scanning Centre (U.K.), <http://www.publichealth.bham.ac.uk/horizon/> |
| NHS QIS | NHS Quality Improvement Scotland (U.K.), http://www.nhshealthquality.org/nhsqis/nhsqis_sub_publications.jsp |
| NICE | National Institute for Clinical Excellence (U.K.), <http://www.nice.org.uk/> |
| NQMC | National Quality Measures Clearinghouse (U.S.A.), [http://www.qualitymeasures.ahrq.gov](http://www.qualitymeasures.ahrq.gov/) |

Table W1: List of websites consulted in hand searches: International Organizations (cont.)

| Abbreviation | Name and Country of the Institution, Internet-Address |
| --- | --- |
| NZGG | New Zealand Guideline Group (New-Zealand), [http://nzgg.org.nz](http://nzgg.org.nz/) |
| NZHTA | New Zealand Health Technology Assessment (New-Zealand), <http://nzhta.chmeds.ac.nz/> |
| SBU | The Swedish Council on Technology Assessment in Health Care (Sweden), <http://www.sbu.se/www/index.asp> |
| SMM | Senter for medisinsk metodevurdering (Norway), <http://www.sintef.no/smm/Publications/Engsmdrag/FramesetPublications.htm> |
| SNHTA | Swiss Network of Health Technology Assessment (Switzerland), [http://www.snhta.ch](http://www.snhta.ch/) |
| TA-SWISS | Zentrum für Technikfolgenabschätzung (Switzerland), <http://www.ta-swiss.ch/framesets/home-d.htm> |
| TNO | Nederlandse Organisatie voor toegepast-natuurwetenschappelijk onderzozoek, TNO (Netherland), <http://www.tno.nl/homepage.html> |
| USPSTF | US Preventive Task Force (U.S.A.), <http://www.ahrq.gov/clinic/uspstfix.htm> |
| VATAP | VA Technology Assessment Program, Department of Veterans Affairs (U.S.A.), <http://www.va.gov/vatap/publications.htm> |
| WVVH | Wetenschappelijke Vereniging van Vlaamse Huisartsen (Belgium), [http://www.wvvh.be](http://www.wvvh.be/) |
| ZonMw | Netherlands Organization for Health Research and Development (Netherlands), <http://www.zonmw.nl/index.asp?s=4535> |

*Table W2: List of websites consulted in hand searches: National Organizations (Germany)*

| Abbreviation | Name of the Institution, Internet-Address |
| --- | --- |
| ÄZQ | Ärztliches Zentrum für Qualität in der Medizin [aqumed - The German Agency for Quality in Medicine] [http://www.aezq.de](http://www.aezq.de/) Guidelines: <http://www.leitlinien.de/leitlinienthemen/index/view?show=11> |
| AWMF | Arbeitsgemeinschaft der Wissenschaftlichen Medizinischen Fachgesellschaften [Association of the Scientific Medical Societies in Germany] <http://www.uni-duesseldorf.de/WWW/AWMF/qs/qs_list.htm> |
| DAHTA@DIMDI | Deutsches Institut für Dokumentation und Information [The German Institute of Medical Documentation and Information] [http://www.dimdi.de](http://www.dimdi.de/) |
| DEGAM | Deutsche Gesellschaft für Allgemein- und Familienmedizin [The German Society of General Practice and Family Medicine] <http://www.degam.de/> |
| DGIM | Deutsche Gesellschaft für Innere Medizin e.V. [The German Society for Internal Medicine] <http://www.dgim.de/> |
| DGK | Deutsche Gesellschaft für Kardiologie – Herz- und Kreislaufforschung [The German Cardiac Society] <http://www.dgk.org/> |
| KBV | Kassenärztliche Bundesvereinigung [The National Association of Statutory Health Insurance Physicians], HTA-Reports: <http://www.kbv.de/hta/hta.htm> |

Table W3: Checklist by the German Working Group on Health Technology Assessment: an example for systematic reviews with/without meta-analyses

| Title: A Systematic Review of the Diagnostic Accuracy of Natriuretic Peptides for Heart Failure.  Authors: Doust JA, Glasziou PP, Pietrzak E, Dobson AJ.  Source: Arch Int Med 2004; 164: 1978-1984. | | | | |
| --- | --- | --- | --- | --- |
| The Document Contents:  Qualitative Information Synthesis Quantitative Information Synthesis X | | | | |
| Class | A. Objective | Yes | No | ? |
| QA | Is the objective relevant? | x |  |  |
| Class | B. Literature Search |  |  |  |
|  | Documentation of the Literature Search: |  |  |  |
| QA | Were the sources documented? | x |  |  |
| QB | Were the search strategies documented? |  |  | x |
| QB | Were the inclusion criteria defined? | x |  |  |
| QB | Were the exclusion criteria defined? |  | x |  |
| Class | C. Appraisal of the Information |  |  |  |
|  | Was the appraisal of the information documented? | x |  |  |
| QA | Were validity criteria considered? | x |  |  |
| QB | Was the appraisal conducted by two independent reviewers? | x |  |  |
| QC | Were the excluded studies documented with reason for preclusion? |  | x |  |
| QC | Was the data extraction documented? | x |  |  |
| QC | Was the data extraction conducted by two independent reviewers? | x |  |  |
| Class | D. Information Synthesis |  |  |  |
|  | Quantitative Information Synthesis: |  |  |  |
| QA | Was the model used for meta-analysis reported? | x |  |  |
| QB | Were tests on heterogeneity conducted? | x |  |  |
| QC | Were sensitivity analyses conducted to test the results of the meta-analyses on their robustness? |  | x |  |
|  | Qualitative Information Synthesis: |  |  |  |
| QA | Was the information synthesis documented comprehensible? |  |  |  |
| QB | Did the authors appraise the evidence? |  |  |  |
| Class | E. Conclusions |  |  |  |
| QB | Were the objectives answered? | X |  |  |
| QB | Did the conclusions follow the findings consistently? | X |  |  |
| QA | Were the methodological limitations of the findings appraised and critical discussed? | X |  |  |
| I | Were recommendations formulated? | X |  |  |
| I | Was a grading of recommendations used? |  | X |  |
| I | Were needs for further research identified? | X |  |  |
| I | Did the document contain information on an update process? |  | x |  |
| Class | F. External Validity of International Results and Conclusions |  |  |  |
|  | Are there differences with regard to the following: |  |  |  |
|  | Epidemiology of the target condition? |  |  | x |
|  | Development status of the technology? |  | x |  |
|  | Indication for use of the technology? | x |  |  |
|  | Target context concerning the target setting and the process of care? |  |  | x |
|  | Financial reimbursement? |  |  | x |
|  | Socio-economic consequences? |  |  | x |
|  | Preferences of patients and providers? |  |  | x |
| Final Assessment: Included | | | | |

**Legend:** Translation in English of the original checklists [44] by the authors of the paper on SGR.

Table W4: List of excluded guidelines with the reason for preclusion

| Guideline (Source) | Reason for Preclusion |
| --- | --- |
| 1. **ABFP 2001**: American Board of Family Practice (2001) Chro­nic Heart Failure. [http://www.familypractice.com/references/ guidesframe.htm](http://www.familypractice.com/references/%0Bguidesframe.htm) | Guideline not evidence based* |
| 2. **ACC 1999**: American College of Cardiology (1999) Consen­sus re­commendations for the management of chronic heart fail­ure. American Journal of Cardiology 83;1A-38A | Guideline not updated since 2000 |
| 3. **AHCPR 1996**: Heart Failure: Evaluation and Care of Patients With Left-Ventricular Systolic Dysfunction. Clinical Practice Guideline Number 11. AHCPR Publication No. 94-0612. <http://hstat.nlm.nih.gov/hq/Hquest/db/local.arahcpr.arclin.lvdc/screen>/TocDisplay/da/1/s/47037/action/Toc;jsessionid=9D9CAB3AC792D15C6A79A780B204AB37  Living With Heart Disease: Is It Heart Failure? Consumer Guide­line Number 11. AHCPR Publication No. 94-0614. National Library of Medicine DOCLINE Information: CAT/9436359 http://hstat.nlm.nih.gov/hq/Hquest/db/local.arahcpr.aruser.lvdp/screen/TocDisplay/s/47037/action/Toc | Guideline not updated since 2000 |
| 4. **AMDA 2002**: American Medical Directors Association (2002) Heart Failure. Clinical Practice Guidelines No. 6. <http://www.amda.com//info/cpg/heartfailure.htm> | Guideline not evidence based* |
| 5. **BCC 2001**: Brisbane Cardiac Consortium. (2001) Clinical Practice Guideline: Hospital management of congestive heart failure. [http://www.health.qld.gov.au/bcc/guidelines/CHF%20 HospGuidelines2%281%29.pdf](http://www.health.qld.gov.au/bcc/guidelines/CHF %0BHospGuidelines2(1).pdf), [http://www.health.qld.gov.au/ bcc/clinical_practice.htm](http://www.health.qld.gov.au/%0Bbcc/clinical_practice.htm) | Guideline exclusively for inpatient care |
| 6. **Brito 2001**: Brito D (2001) [Guidelines for the diagnosis and treat­ment of chronic heart failure] Rev Port Cardiol 20(10): 1049-51 | Guideline in Portuguese |
| 7. **GICRP 2003**: Task Force per le Attivita di Psicologia in Car­diologia Riabilitativa e Preventiva, Gruppo Italiano di Cardiologia Riabilitativa e Preventiva (2003) [Guidelines for psychology activities in cardiologic rehabilitation and prevention]. Monaldi Arch Chest Dis 60(3): 184-234 | Guideline in Italian |
| 8. **HFSA 1999**: Heart Failure Society of America (1999) HFSA guide­lines for management of patients with heart failure caused by left ventricular systolic dysfunction: Pharmacological ap­proa­ches. <http://www.hfsa.org/pdf/lvsd_heart_failure.pdf> | Guideline not updated since 2000 |
| 9. **KPNC 2000**: Kaiser Permanente Northern California (2000) Clinical Practice Guideline For Heart Failure Due To Left-Ven­tricular Systolic Dysfunction. [http://www.kaiserpapers.org/left ven.html](http://www.kaiserpapers.org/left%0Bven.html) | Guideline not evidence based* |
| 10. **MSSM 2003**: Mount Sinai School of Medicine: Galin I & Baran DA (2003) Congestive Heart Failure: Guidelines for the Primary Care Physician. The Mount Sinai Journal Of Medicine 70(4): 251 – 264  Und: Gomberg-Maitland M, Baran DA, Fuster V (2001) Treat­ment of Congestive Heart Failure. Guidelines for the Primary Care Physician and the Heart Failure Specialist. Arch Intern Med 161: 342 – 352 | Guideline not evidence based* |
| 11. **Navazio 2000**: Navazio F (2000) [New guidelines in the treatment of congestive heart failure] Clin Ter 151(3):179-81 | Guideline in Italian |

Table W4: List of excluded guidelines with the reason for preclusion (cont.)

| Guideline (Source) | Reason for Preclusion |
| --- | --- |
| 12. **NHF/NZ 1997**: National Heart Foundation of New Zealand (1997) New Zealand guidelines for the management of chronic heart failure NZ Med J 99-107 | Guideline outdated – new version included |
| 13. **NHG 1995**. Walma EP, Bakx HCA, Besselink RAM, Ham­stra PWJ, Hendrick JMA, Koote JHA, van Veelen AWC, Vink R und Geijer RMM (1995) NHG-Standaard Hartfalen. M51. <http://nhg.artsennet.nl/upload/104/standaarden/M51/start.htm> | Guideline in Dutch, not evidence based*, not updated since 2000 |
| 14. **Ohkusa 2003**: Ohkusa T, Matsuzaki M (2003) [Guidelines for the evaluation and management of chronic heart failure] Nippon Rinsho 61(5):723-30. Und: Matsuzaki M, Okusa T (2002) [Practice guidelines for patients with chronic heart failure] Nippon Naika Gakkai Zasshi 10; 91(3):973-90. | Guideline in Japanese |
| 15. **PRODIGY 2004**: PRODIGY Guidance – Heart failure. [http://www](http://www/).prodigy.nhs.uk/guidance.asp?gt=Heart%20Failure Patient Information Leaflet Heart Failure. [http://www.prodigy.nhs.uk/clinicalguidance/releasedguidance/webBrowser/pils/PL83.htm](http://www.prodigy.nhs.uk/clinicalguidance/releasedguidance/web%0BBrowser/pils/PL83.htm) | Guideline not evidence based* |
| 16. **SAMA 1998**: South African Medical Association Heart Failure Working Group. Heart failure clinical guideline. South African Medical Journal 1998;88:1135-55. | Guideline not updated since 2000 |
| 17. **SGK 2002**: Schweizerische Gesellschaft für Kardiologie. Empfehlungen zur Diagnose und Behandlung der chronischen Herzinsuffizienz. Schweizerische Ärztezeitung 2002;1233-42. | Adapted Guideline from ESC 2001 |
| 18. **SIGN 1999**: Scottish Intercollegiate Guidelines Network. Diagnosis and treatment of heart failure due to left ventricular _ystolic dysfunction. <http://www.sign.ac.uk/pdf/sign35.pdf>, <http://www.sign.ac.uk/pdf/qrg35.pdf>, <http://www.sign.ac.uk/patients/index.html> | Guideline not updated since 2000 |
| 19. **Thilly 2003** : Thilly N, Zannad F, Dufay E, Juilliere Y, Briancon S; Service d’Epidemiologie et Evaluation Cliniques (UPRES EA1124), Hopital Marin, Nancy, France. [Angiotensin-converting enzyme inhibitors in congestive heart failure: clinical practice guidelines] Therapie. 2003; 58(4): 341-9. | Guideline in French |
| 20. **UMHS 1999**: University of Michigan Health System. Heart failure: systolic dysfunction, Guidelines for clinical care. <http://www.cme.med.umich.edu/pdf/guideline/heart.pdf> | Guideline not updated since 2000 |
| 21. **VPQ 1999**: Vermont Program for Quality in Health Care. Heart failure in Vermont. [http://www.vpqhc.org](http://www.vpqhc.org/) | Guideline not updated since 2000 |
| 22. **WHO 1995**: World Health Organization. Concise guide to the management of heart failure. A patient's guide to control of heart failure. [http://www.who.int/cardiovascular_diseases /resources/publications/en/print.html](http://www.who.int/cardiovascular_diseases%0B/resources/publications/en/print.html) | Guideline not updated since 2000 |

**Legend:** *Criteria for “evidence based” guidelines: the guideline provides information about the search methods, the selection of evidence, and the methods used to formulate the recommendations. In addition, the key recommendations (or most of the recommendations) are explicitly linked to the supporting evidence.

Table W5: Results of Consistency Analyses: List of Type-3-Consistencies and Type-A-Inconsistencies

| Consistent Recommendations of the included guidelines based on weak evidence / expert opinion (Type-(3)-Consistencies:   - Symptoms and signs of chronic heart failure - Laboratory tests in diagnosing chronic heart failure - 12 lead electrocardiogram (ECG) - Chest X-ray - Echocardiography - Life-style modification - Vaccination - No ARBs instead of ACE inhibitors in ACE inhibitor-naïve patients - Oral anticoagulation for patients with sinus rhythm or for those with a history of thromboembolism, left ventricular aneurysm, or intracardiac thrombus - Antiplatelet agents in patients with concomitant coronary artery disease - Pharmacological therapy of diastolic heart failure |
| --- |
| Major Inconsistencies of Recommendations (Type-(A)-Inconsistencies):   - BNP- and NT-proBNP-test in diagnosing heart failure - ARBs additional to ACE inhibitors and beta-blockers (triple therapy) - ARBs additional to ACE inhibitors in those patients, who have contra-indications for or can not tolerate beta-blockers |

Table W6: Comparison of different quality appraisals for the included guidelines

|  | Muth et al.  Median Target and Result Score* | AeZQ  Total Score, absolut (0-40)^†^ | AeZQ  Total Score, standardized^††^ |
| --- | --- | --- | --- |
| ACC/AHA 2001 [50] | 0,54 | 18 | 0,45 |
| AKDAE 2001 [45] | 0,27 | 26 | 0,65 |
| CCS 2001, 2002/2003 [55] | 0,33 | 21 | 0,53 |
| DGK 2001 [46] | 0,19 | 16 | 0,40 |
| DIeM 2003/2004 [47] | 0,35 | ‑ | ‑ |
| Duodecim 2004 [60] | 0,39 | 14 | 0,35 |
| DVA 2002 [51] | 0,63 | 27 | 0,68 |
| ESC 2001/2002 [62] | 0,32 | 19 | 0,48 |
| ISCI 2003 vs. 2004^*^ [52] | 0,70 | 25 | 0,63 |
| LLGH 2003 [48] | 0,58 | ‑ | ‑ |
| NHF/Austr. & CSANZ 2002 [58] | 0,31 | 23 | 0,58 |
| NHF/NZ 2001 [59] | 0,35 | 23 | 0,58 |
| NICE 2003 [61] | 0,71 | 33 | 0,83 |
| OPOT 2000 [57] | 0,31 | 22 | 0,55 |
| UM 2001 [53, 54] | 0,33 | 16 | 0,40 |
| UWH 2001 [49] | 0,21 | ‑ | ‑ |

**Legend:** Instruments used for the appraisal of guideline quality based on the same dimensions but used a different scoring and had to be standardized, therefore. ^*^See text; ^†^Data from AeZQ 2005[[1]](#endnote-2), ^††^Calculated as a percentage of the maximum possible score

1. Fessler J, Gross J, Hoepp HW et al. (Autoren), Thole H (Ed.), Ärztliches Zentrum für Qualität in der Medizin (ÄZQ), Leitlinien-Clearingstelle (Hrsg.) (2005) Leitlinien-Clearingbericht "Herzinsuffizienz": Leitlinien-Clearingverfahren von Bundesärztekammer und Kassenärztlicher Bundesvereinigung in Kooperation mit Deutscher Krankenhausgesellschaft, Spitzenverbänden der Krankenversicherungen und gesetzlicher Rentenversicherung. Niebuell: Verl. Videel, (AeZQ-Schriftenreihe; Bd. 18) ISBN 3-89906-909-9 [↑](#endnote-ref-2)
